# Child with Deletion 9p Syndrome Presenting with Craniofacial Dysmorphism, Developmental Delay, and Multiple Congenital Malformations

**DOI:** 10.1155/2013/785830

**Published:** 2013-07-25

**Authors:** Nirmala D. Sirisena, U. Kalpani S. Wijetunge, Ramya de Silva, Vajira H. W. Dissanayake

**Affiliations:** ^1^Human Genetics Unit, Faculty of Medicine, University of Colombo, Kynsey Road, Colombo 08, 00800, Sri Lanka; ^2^Asiri Center for Genomic and Regenerative Medicine, Asiri Surgical Hospital, Colombo 05, 00500, Sri Lanka; ^3^Lady Ridgeway Children's Hospital, Colombo 08, 00800, Sri Lanka

## Abstract

A 4-month-old Sri Lankan male child case with a *de novo* terminal deletion in the p22→pter region of chromosome 9 is described. The child presented with craniofacial dysmorphism, developmental delay, and congenital malformations in agreement with the consensus phenotype. A distinctive feature observed in this child was complete collapse of the left lung due to malformation of lung tissue. Cytogenetic studies confirmed terminal deletion of the short arm of chromosome 9 distal to band p22 [46,XY,del(9)(p22→pter)]. This is the first reported case of a *de novo* deletion 9p syndrome associated with pulmonary hypoplasia. This finding contributes to the widening of the spectrum of phenotypic features associated with deletion 9p syndrome.

## 1. Introduction

Deletion 9p syndrome is a rare structural chromosomal disorder characterized by craniofacial dysmorphism, various congenital malformations, and psychomotor delay. The consensus phenotype consists of trigonocephaly with prominent forehead, small palpebral fissures, flat nasal bridge, low-set dysplastic ears, anteverted nostrils, long philtrum, and disproportionately long phalanges [1]. Congenital malformations include cardiac defects, inguinal hernia, omphalocele, and abnormal external genitalia [2]. These features are known to be characteristically associated with deletion 9p syndrome, and knowledge of these associations aids in the early clinical recognition and cytogenetic diagnosis of this syndrome [3]. The breakpoints usually occur in bands from 9p22 to 24, and most patients have either pure terminal deletions involving 9p or unbalanced chromosomal rearrangements involving chromosome 9p and another chromosome. The deletion is *de novo* (sporadic) in two-thirds of cases arising during either paternal or maternal meiosis and familial in the remaining one-third arising from unbalanced chromosome rearrangements inherited from a parent with a balanced translocation [1, 4]. The first case was reported by Alfi et al. in 1973, and since then, more than 150 cases have so far been reported worldwide [1, 3]. This paper describes the first reported case of a *de novo* deletion 9p syndrome associated with pulmonary hypoplasia. 

## 2. Case Presentation

A 4-month-old Sri Lankan male child with dysmorphic features and congenital malformations was referred with a clinical suspicion of Down syndrome to our centre for chromosomal analysis and genetic counseling. He was the first child born to healthy, nonconsanguineous parents. Family history was unremarkable. The father was aged 35 years and the mother 30 years at the time of baby's birth. All the antenatal scans were reported to be normal. The mother presented with premature rupture of membranes and pyrexia at 33 weeks of gestation, and the baby was delivered preterm by an emergency lower segment caesarean section due to fetal distress. At birth, he weighed 2.175 kg (50th centile) and was found to have craniofacial dysmorphism along with an omphalocele. His Apgar score was 8, 9, and 10 at 1, 5, and 10 minutes, respectively. Surgical repair of omphalocele was performed on the second day of life, and the baby was managed postoperatively at the surgical intensive care unit on a ventilator for 48 days due to respiratory distress and repeated failed extubations. He was also concurrently treated for neonatal sepsis and was discharged home after full recovery.

**Figure 1:**
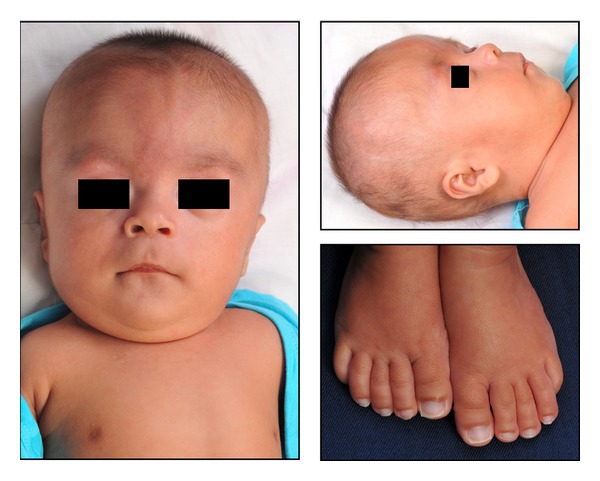
Frontal and lateral views of the face along with the feet showing long toes and square nails at the age of 4 months.

On examination at the age of 4 months, the child's weight, length, and occipitofrontal circumference (OFC) were 3.4 kg (<3rd centile), 57 cm (<3rd centile), and 37 cm (<3rd centile). His gross motor and social milestones were delayed. Speech development could not be assessed due to his age. He had multiple craniofacial dysmorphic features such as prominent metopic ridge with trigonocephalic head and flat occiput, midfacial hypoplasia, bilateral epicanthal folds, high arched eyebrows, downslanting palpebral fissures, ocular hypertelorism, hypoplastic supraorbital ridges, flat nasal bridge, anteverted nostrils,  low set ears with malformed auricles, long smooth philtrum with thin upper lip, downturned corners of the mouth, micrognathia, short neck, asymmetrically flattened chest wall on the left side with wide-set nipples, and long fingers and toes due to increased length of the middle phalanges. The nails were square in shape (Figure 1).

Echocardiogram showed osteum secundum atrial septal defect, and high resolution computed tomography scan (HRCT) of the lungs showed a complete collapse of the left lung with almost absent lung tissue. The right lung was hyperexpanded with midline shift to the left side. There was right lower lobe atelectasis, mild interstitial thickening, and herniation of part of the right lobe to the left side but no evidence of brochopulmonary dysplasia or chronic lung disease. Trachea and major bronchi were normal in caliber, and pleural space was clear. Additional laboratory investigations, including blood urea, serum electrolytes, creatinine, alkaline phosphatase, calcium, phosphorus, and thyroid profiles, were all normal. 

**Figure 2:**
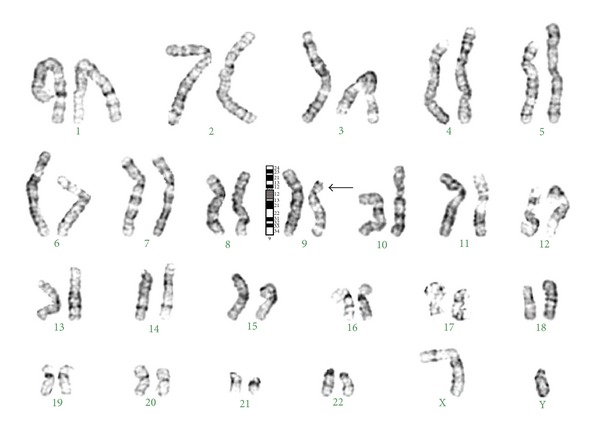
Karyogram showing terminal deletion in the short arm of chromosome 9 [46,XY,del(9)(p22→pter)].

Chromosome culture and karyotyping of the child and the parents were performed on routinely cultured peripheral blood lymphocytes. A total of 30 metaphase spreads were analyzed in each case according to conventional GTG banding technique. The maximum banding resolution achieved was 500 bands. All the metaphases from the child showed terminal deletion of the short arm of chromosome 9 distal to band p22 as the sole abnormality. His karyotype was 46,XY,del(9)(p22→pter) shown in Figure 2, and normal karyotypes were seen in both parents.

## 3. Discussion

The phenotypic features observed in this child are in line with the consensus phenotype observed in other deletion 9p syndrome patients reported in the scientific literature [1, 4–6]. In addition, this child had features of pulmonary hypoplasia which has not previously been reported in patients with *de novo* 9p deletion syndrome. It is assumed that this feature may be a novel phenotype associated with the disruption of yet unidentified genes in this region which may be involved in pulmonary development. 

Numerous reports in the scientific literature have observed that deletion 9p syndrome is caused by a partial monosomy due to deletion of genetic material from the short arm of chromosome 9 with breakpoint sites mainly in the regions 9p21 to 9p24 [1, 4, 5, 7, 8]. The 9p22→pter breakpoint observed in this child is consistent with that described in the literature. High resolution mapping of the breakpoints to delineate the exact size of the deleted segment and exclusion of cryptic chromosome rearrangements however could not be performed in this case using fluorescence in situ hybridization (FISH) or microarray-based comparative genomic hybridization (aCGH) since these techniques have not yet been established at the two genetic diagnostic centres in the country. 

A number of previous studies had delineated a proposed critical region for deletion 9p phenotype to a 4 to 6 Mb region in 9p22.2-p23 [8, 9]. Faas et al. further refined the critical region to a 3.5 Mb interval on 9p, a region containing at least seven known genes: *FREM1 (FRAS1 related extracellular matrix 1), CER1 (cerberus 1 homolog), ZDHHC21 (zinc finger, DHHC-type containing 21), NFIB (nuclear factor I/B), MPDZ (multiple PDZ domain protein), LURAP1L (leucine rich adaptor protein 1-like),* and *TYRP1 (tyrosinase-related protein 1)* [9]. Genes within this region are thought to contribute to some of the cardinal features of deletion 9p syndrome. It is hypothesized that defects in the *CER1* gene may play a role in the trigonocephaly phenotype [10], but several recent studies have disproved this [2, 9]. 

In an attempt to develop genotype/phenotype correlations, Hauge et al. mapped the breakpoints of 10 patients with deletion 9p using FISH and microarray analyses. They observed that the minimal deleted region shared by their patients with clinically relevant phenotypes included the first 2 Mb of 9 pter [3]. However, Huret et al. reviewed 80 cases of deletion 9p syndrome and observed that dysmorphic and developmental features are typical and do not seem to differ with the breakpoint or the size of the deleted segment [7]. Clear-cut genotype/phenotype correlations have therefore not been established mainly as a result of significant phenotypic diversity among deletion 9p syndrome patients and insufficient fine mapping data [2, 3]. Highly refined genetic characterization combined with detailed clinical description of several more patients with this syndrome is therefore needed for identifying the molecular mechanisms that lead to each characteristic of the phenotype. Gonadal dysgenesis with sex reversal, hypospadias, cryptorchidism, and malformed external genitalia have also been reported in several previous cases of deletion 9p syndrome [2, 4]. It is postulated that haploinsufficiency of sex determining genes, *DMRT1 *and* 2*, located on 9p24.3 together with other genetic defects may be responsible for this phenotype. Genital abnormalities were however not observed in this child. Previous reports of patients with 9p24 deletions and normal male external genitalia and/or very mild gonadal abnormalities have been described [11]. This variable penetrance may be attributed to the interaction of various other genetic modifiers on the phenotype. 

In conclusion, the phenotypic features observed in this child add to the spectrum of clinical features seen in patients with deletion 9p syndrome. The presence of craniofacial dysmorphism, trigonocephaly, prominent forehead, anteverted nostrils with long philtrum, psychomotor retardation, and congenital malformations such as omphalocele and cardiopulmonary anomalies should alert clinicians to the possibility of deletion 9p syndrome and refer such patients for cytogenetic confirmation. Cytogenetic analysis is vital as it aids in the precise and early diagnosis of this syndrome and helps to exclude other causes and provide appropriate genetic counseling.
